# Influence of Sugar Beet Pulp Supplementation on Pigs’ Health and Production Quality

**DOI:** 10.3390/ani12162041

**Published:** 2022-08-11

**Authors:** Sarunas Badaras, Dovile Klupsaite, Modestas Ruzauskas, Romas Gruzauskas, Egle Zokaityte, Vytaute Starkute, Ernestas Mockus, Jolita Klementaviciute, Darius Cernauskas, Agila Dauksiene, Laurynas Vadopalas, Elena Bartkiene

**Affiliations:** 1 Institute of Animal Rearing Technologies, Faculty of Animal Sciences, Lithuanian University of Health Sciences, Mickeviciaus Str. 9, LT-44307 Kaunas, Lithuania; 2 Institute of Microbiology and Virology, Faculty of Veterinary Medicine, Lithuanian University of Health Sciences, Mickeviciaus Str. 9, LT-44307 Kaunas, Lithuania; 3 Department of Anatomy and Physiology, Faculty of Veterinary Medicine, Lithuanian University of Health Sciences, Mickeviciaus Str. 9, LT-44307 Kaunas, Lithuania; 4 Department of Food Science and Technology, Kaunas University of Technology, Radvilenu Rd. 19, LT-50254 Kaunas, Lithuania; 5 Department of Food Safety and Quality, Faculty of Veterinary Medicine, Lithuanian University of Health Sciences, Mickeviciaus Str. 9, LT-44307 Kaunas, Lithuania

**Keywords:** pig health, faeces microbial profile, sugar beet pulp, carcass classes, pork quality, emotions

## Abstract

**Simple Summary:**

Fibre inclusion in animals’ diets can improve digestive health and protect the animal against diarrhoea. However, studies have shown that fibrous feedstuffs can have a variable effect on pig growth, health and production quality. Sugar beet pulp (SBP) is a cheap fibrous material which contains more lysine than wheat grain and has a similar gross energy and crude protein content compared to corn grains. Nevertheless, the effect of fibre supplementation in the diet, especially using SBP, on pork quality has not been widely reported. This study evaluated the effect of an SBP-supplemented diet (3%) on Large White/Norwegian Landrace piglets’ growth performance, health parameters (blood characteristics and faeces microbial profile), carcass and meat quality. The tested diet reduced the average daily gain but improved the carcass quality. It also affected most of the blood parameters, the microbial profiles in pig faeces and the fatty acid and volatile compound profiles of pork meat. Higher drip loss, protein content and redness, along with a lower cooking loss, intramuscular fat content and lightness, were observed in the meat from pigs fed with SBP. Most of the sensory properties, as well as the overall acceptability, were more highly rated for this meat. The SBP-supplemented diet could be beneficial for the improvement of pigs’ gut health and pork quality. However, further studies are needed to indicate which compounds of the SBP dietary fiber are responsible for these desirable changes.

**Abstract:**

Fibrous feedstuffs can have a variable effect on pig growth, health and meat quality. The effect of sugar beet pulp (SBP) supplementation in the diet on pork quality has not been widely reported. This study examines the effect of an SBP-supplemented (3%) diet (TG-I group) on 300 Large White/Norwegian Landrace pigs in terms of growth performance, blood parameters, microbial profiling of faeces, carcass parameters and meat quality, including the profiles of biogenic amines (BAs), fatty acids (FAs) and volatile compounds (VCs). After 163 days of the experiment, TG-I pigs had a significantly lower average daily gain and feed conversion ratio than pigs in the control group, as well as a significantly higher percentage of carcasses in the S and KN classes and a lower percentage in the E and U classes (*p* ≤ 0.05). Faeces of TG-I contained significantly more bacteria that are considered probiotic. Significant differences (*p* ≤ 0.05) were found in most of the blood parameters, FA, VC profile and emotional responses between the two groups. Higher drip loss, protein content and redness, as well as lower cooking loss, intramuscular fat content and lightness were observed in the meat of TG-I. Most of the sensory properties, as well as overall acceptability, were rated higher for the meat of TG-I. Based on the results, a diet containing 3% of SBP could be beneficial for the improvement of pigs’ gut health and pork quality. However, further studies are needed to indicate which compounds of the SBP dietary fiber are responsible for these desirable changes.

## 1. Introduction

Feed usually contains ingredients from different countries and production systems [1,2,3]. Whilst some of them may consist of simple supply chains, others may involve many transactions between farmers and producers, and the total number of transactions related to a single product is likely to be high [4,5]. Local food supply chains are often considered to be sufficiently sustainable, mainly because they support mixed and organic farming and reduce emissions and the externalities of long-distance transport [6,7]. Local food supply chains are also valued for their ability to create rural businesses and build rural communities by dismantling agribusiness monopolies and creating links between man and nature [8,9].

Rising cereal flour prices are forcing pork producers to look for alternative feed materials [10,11]. Many alternative feed materials contain more fibre than the traditional feed stock they replace; therefore, their inclusion increases the amount of dietary fibre [12,13]. Increased dietary fibre is a concern because the ability of young pigs to digest fibre is limited [14,15]. Sugar beet pulp (SBP), which is the co-product of sugar beet processing, with a global production of up to 189 mln tons in 2021/2022, is a fibre-rich feed [16]. It should be noted that SBP contains similar gross energy and crude protein contents compared to corn grains [17]. Additionally, SBP contains fibre that is fermentable, has a high water-binding capacity and can alter the physical and chemical properties of the digestive tract, thus affecting feed intake and animal growth [18,19,20]. The fermentation of dietary fibre, such as SBP, in the gut promotes the multiplication of bacterial populations such as those of *Lactobacillus* and *Bifidobacterium*, which are generally considered to be beneficial to intestinal health, in part by preventing colonisation by opportunistic pathogens [20,21]. A lower risk of gastric ulcers has also been observed, as well as improved colonic mucosal integrity [22]. All of this helps to strengthen the digestive system and prevent diarrhoea [5]. Therefore, the promotion of gastrointestinal health is expected to improve performance. Conversely, more cases of diarrhoea and colitis have sometimes been reported in weaned piglets, such as in the cases of soluble fibre supplements that increase gastrointestinal viscosity [23]. Some authors have observed that the dilution of energy in fibrous diets, faster intestinal motility and decreased ingestion due to early satiety have impaired productivity [24]. Additionally, diets rich in dietary fibre can have a positive effect on pig intestinal health and meat quality [25,26]. As with intestinal health, the effects on workability may vary depending on the origin and type of fibre [27]. Dietary fibre components are not digested by endogenous digestive enzymes and are therefore the main substrates for bacterial fermentation in the distal intestine [28]. The main fermentation products are short-chain organic acids, mainly lactate, acetate, propionate and butyrate [29]. Organic acids have been suggested to stimulate gastrointestinal growth by promoting epithelial cell proliferation [30]. In an acidic environment, organic acids can inhibit the growth of intestinal bacterial pathogens such as *Salmonella*, *E. coli* and *Clostridium species* [31]. Changes in the intestinal microflora can affect the technological composition of pork meat, and its profile of volatile compounds (VC) and organoleptic properties can change, which can lead to the acceptability of the organoleptic properties of meat [32,33].

Although a considerable number of studies have analysed the influence of a fibrous diet on animal health and performance, the reported findings are contradictory due to different experimental conditions, differences in animal age and variations in the content and type of fibre source in the diet. Moreover, the effect of a fibrous diet, especially with SBP, on pork quality has not been comprehensively examined. Thus, this study evaluated the effect of an SBP-supplemented diet on pig growth performance, health parameters (blood characteristics and faeces microbial profile), carcass and meat quality, including the profiles of biogenic amines (BA), fatty acids (FA) and VC.

## 2. Materials and Methods

### 2.1. Sugar Beet Pulp Used for Pig Feeding 

Sugar beet pulp (dried) was obtained from the company Imlitex Agro (Kaunas, Lithuania) and had the following composition: moisture—12.00%, crude protein—10.41%, crude fat—0.85%, crude fibre—17.82%, calcium—1.34%, phosphorus—0.12%.

### 2.2. Animals and Housing

The study was performed at a pig farm in the Klaipeda District (Kantvainu Village, Lithuania) and at the Institute of Animal Rearing Technologies, Lithuanian University of Health Sciences (Kaunas, Lithuania). An experiment was conducted using 300 81-day-old Large White/Norwegian Landrace (LW/NL) pigs (150 pigs in each group, 14–15 pigs per pen and 42–44 pigs per cage). The trial started with pigs having an initial body weight of 29.1–32.4 kg in both (control and treatment) groups. During the first fattening period, the pigs were kept in a pen consisting of 90% concrete grades and 10% heated (33 °C) concrete floors. The barn was heated only in the first weeks after the pigs had been moved in. In the second fattening period, the pigs were kept in a cage with a concrete grade without heating. The barn was preheated up to a temperature of 19 °C by diesel heaters before the animals were moved in. During the fattening period, both barns were optimally ventilated. Drinking water was available *ad libitum* throughout the trial by nipple drinkers. Antibiotic treatment was not applied.

### 2.3. Experimental Design and Diets 

The pigs (81 days old) were distributed into two groups at the beginning of the experiment, namely (i) a basal diet groups (control group—CG) and (ii) basal diet with dried SBP group (treated group—TG-I). The basal feed was formulated according to the Nutrient Requirements of Swine [17]. The feed composition and nutritional values are shown in Table 1. Dietary contents were analysed according to the Association of Official Agricultural Chemists (AOAC) recommendations [34]. Other nutritional value parameters, including ME, were calculated by using a feed optimization program and tables of composition and nutritional value of feed materials.

All animal groups were fed with wet feed (water and feed ratio 3/1); the equipment used for feeding was WEDA (Dammann & Westerkamp GmbH, Goldenstedt, Germany).

The pigs’ growth performance was evaluated by testing all pigs from both groups. For the evaluation of blood parameters, 15 pigs from each group were randomly selected. For the microbial profiling of faeces, samples from 15 randomly selected pigs were taken before the distribution of pigs into two groups (day 81), and 15 samples from each group were taken after the feeding experiment on day 163.

### 2.4. Pigs’ Growth Performance 

Group body weight (BW) gain was recorded on days 81, 116, and 163 of age using an electronic weighing system (model type: IT1000, SysTec GmbH, Bergheim, Germany). To weigh the whole group, the complete pig pens were driven out into the central corridor, where the pigs were immediately weighted.

The feed conversion ratio (FCR) was calculated from feed intake (87% of dry matter) and BW gain, recorded on the same days, using a WEDA (Dammann & Westerkamp GmbH, Germany) automated feeding system consisting of a mixer with weight sensors under the mixer and pumps with flow metres. The feed components were mixed in a mixer and fed to the pigs according to the feeding curve. The flowmeter at the time of feeding calculated the amount of feed added to each trough. 

### 2.5. Microbial Profiling Analysis

Faecal samples from each group were pooled to obtain separate representative samples of pigs before the experimental feeding and for CG and TG-I groups after the experiment and kept in DNA/RNA Shield (1:10 dilution; R1100-250, Zymo Research, Irvine, CA, USA) at −70 °C before DNA extraction. The DNA was extracted with a faecal DNA MiniPrep kit (D6010, Zymo Research, USA). Library preparation, metagenomic sequencing and taxonomic characterisation of reads were performed as described previously [35]. The ZymoBIOMICS Microbial Community Standard (D6300, Zymo Research, Murphy Ave, Irvine, CA, USA) was used as a microbiome profiling quality control. The results of the taxonomic classification were visualised using the interactive online platform https://genome-explorer.com (accessed on 24 March 2022). The number of bacterial reads at genus level was compared between CG and TG-1 groups at the end of the experiment.

### 2.6. Blood Analysis

Pigs, with a fixed nose twister, were bled from the jugular vein (at days 81 and 163) into vacuum blood tubes (BD Vacutainer, Plymouth, UK) before the morning feeding. Tubes with clot activator were used for biochemical examination. The parameters included aspartate aminotransferase (AST), alanine aminotransferase (ALT), cholesterol, high-density lipoprotein cholesterol (HDL), low-density lipoprotein (LDL) cholesterol, triglycerides (TG), total protein (TP), albumin (ALB), triiodothyronine (T3), thyroxine (T4), immunoglobulins IgA, IgM, and IgG, glucose (GLU), creatinine (CREA) were analysed by the Jaffe method, alkaline phosphatase (AP), thyroid-stimulating hormone (TSH), total bilirubin (TBI) and urea (UREA). Blood parameters were analysed with an automatic biochemistry analyser in the accredited laboratory “Anteja” (Klaipeda, Lithuania). 

### 2.7. Slaughter and Measurement of Carcass Parameters 

The animals were slaughtered at the Ltd. “Utenos mesa” slaughterhouse (Utena, Lithuania) according to Council Regulation (EC) No. 1099/2009 [35]. Pig carcasses were graded according to EU Regulation No. 1308/2013 [36] and Commission Implementing Decision (EU) 2020/871 [37]. Carcasses were divided into six classes (S, E, U, R, O or P), according to their estimated lean meat content (S > 60%, E = 55–60%, U = 50–55%, R = 45–50%, O = 40–45% and *p* < 40%) at the same slaughterhouse.

### 2.8. Meat Quality Analysis

#### 2.8.1. Evaluation of the Main Meat Quality Parameters

The raw *Longissimus dorsi* muscle (from the 5th to 8th thoracic vertebrae part) from pigs was used for the analysis of meat quality parameters, including BA, FA and VC. Dry matter was measured by Scaltec SMO—01, drying samples at 103 ± 2 °C. Meat pH was evaluated by a pH-metre “Inolab 3”, using a contact electrode [38]. Colour coordinates of the meat were detected by a Minolta Chroma Meter (CR-400, Minolta Camera, Osaka, Japan), measuring L* values of lightness, a* values for redness and b* values for yellowness. Water holding capacity (WHC) was analysed using the filter paper press method. For this, the sample (2 g) was placed on a filter paper (Whatman filter paper 41/ashless), compressed between two plexiglass sheets, and received a pressure exerted by a weight of 1 kg for 10 min. The *drip loss* (DL) was measured as the weight loss during suspension of a standardised (40–50 g and approximately 30 × 60 × 25 mm) muscle sample (in an airtight container over 24 h at 4 °C). Cooking loss (CL) was calculated as the weight difference between the samples (in a plastic bag) before and after cooking in a water bath (internal temperature of 70 °C for 30 min). The intramuscular fat was determined by an automatic system for fat extraction “Soclet SE 416 macro” (Gerhardt, Germany) [39]. The protein content was determined by the Kjeldahl method and the ash content by organic matter incineration at +700 °C [40].

#### 2.8.2. Evaluation of Biogenic Amine Content in Pork Meat

Sample preparation and determination of BA content in pork meat samples were performed according to the method of Ben-Gigirey et al. [41], with some modifications [42]. The BAs were extracted with 0.4 mol/L perchloric acid, and dansyl chloride solution in acetonitrile (10 mg/mL) was used as a derivatisation reagent. The Varian ProStar HPLC system (Varian Corp., Palo Alto, CA, USA) was composed of two ProStar 210 pumps, a ProStar 410 autosampler, a ProStar 325 UV/VIS detector and the Galaxy software (Agilent, Santa Clara, CA, USA) for data processing. For the separation of BAs, a Discovery^®^ HS C18 column (150 × 4.6 mm, 5 µm; SupelcoTM Analytical, Bellefonte, PA, USA) was used. The BAs were identified based on their retention times in comparison to their corresponding standards.

#### 2.8.3. Fatty Acid Composition Analysis

Extraction of lipids for FA analysis was performed with chloroform/methanol (2:1 *v*/*v*), and FA methyl esters (ME) were prepared as described by Pérez-Palacios et al. [43]. The FA composition of pork meat was determined using gas chromatograph GC-2010 Plus (Shimadzu Corp., Kyoto, Japan) equipped with a mass spectrometer GCMS-QP2010 (Shimadzu Corp.). Separation was carried out on an Rxi-5 ms column (30 m length, 0.25 mm ID and 0.25 μm df (Restek, Bellefonte, PA, USA). The mass spectrometer was operated at full scan mode, and the analyte was injected in split mode at a 1:60 split ratio. The carrier gas was helium at a flow rate of 0.91 mL/min. The FAME concentration was determined using a calibration curve, and results were expressed as percentages of the total FAME concentration in the sample. The calibration curve was prepared using the standard Supelco 37 Component FAME Mix (Merck & Co., Inc., Kenilworth, NJ, USA).

#### 2.8.4. Volatile Compound Profile

The VCs of pork meat samples were analysed by gas chromatography-mass spectrometry (GC-MS) as described by Vadopalas et al. [44], with some modifications. A solid-phase microextraction (SPME) device with Stableflex (TM) fibre-coated with a 50-µm DVB-PDMS-Carboxen™ layer (Supelco, Bellefonte, PA, USA) was used for sample preparation. For headspace extraction, 2 g of sample was homogenised with 4 mL of sodium chloride solution (30% *w*/*v*) in a 20 mL extraction vial sealed with polytetrafluoroethylene septa and thermostated at 60 °C for 30 min, exposing the fibre in the headspace. The desorption time was 2 min. Prepared samples were analysed with a GCMS-QP2010 (Shimadzu, Japan) GC-MS, using an Rxi-5 ms capillary column (30 m × 0.25 mm ID × 0.25 µm film thickness) for analysis. The MS was operated at full scan mode (35–500 *m*/*z*), and the following conditions were used for analysis: column flow rate (helium gas, 99.999% purity) 0.95 mL/min, injector temperature 250 °C, ion source temperature 220 °C, interface temperature 280 °C. Sample injection was carried out for 2 min to ensure full desorption of VC from the SPME fibre. A temperature gradient was programmed from start at 40 °C (3 min hold) to 220 °C (7 °C/min) up to 260 °C (10 °C/min) (6 min hold). The VCs were identified according to the mass spectrum libraries (NIST11, NIST11S, FFNSC2).

#### 2.8.5. Evaluation of the Pork Meat Sensory Properties, Overall Acceptability, and Emotions Induced in the Panellists

This evaluation was performed at the Lithuanian University of Health Sciences Sensory laboratory, which is equipped with sensory booths. Meat samples were evaluated by 30 panellists [45]. Before the sensory analysis, the meat was cut to 2 cm squares and cooked at 100 °C in water for 10 min (the salt was not added). The final temperature inside the meat at the end of cooking was 82 °C. Panelists were randomly presented with coded samples under normalised artificial light in test rooms [46] and were instructed to taste one at a time, from left to right. The panelists were provided water between samples. The following sensory properties were evaluated: odour—intensiveness of the overall odour and the extraneous odour, colour—intensiveness of the colour, taste—juiciness, fattiness, softness, intensiveness of the residual taste and overall acceptability of the taste [47]. The intensity of each property was scored on a 5-point linear scale ranging from 0 (“no intensity”) to 5 (“very high intensity”). The overall acceptability was evaluated using a 5-point Likert scale, ranging from 1 (extremely dislike) to 5 (extremely like) [46].

The panellists tasted the presented samples one by one in front of a webcam (Microsoft Corporation, Redmond, WA, USA), and the tasting procedure was recorded [48]. After tasting each sample, the panellist raised one hand and visualised the taste experience with a facial expression. The time for that was not limited. Between samples, panellists were asked to rinse their mouth with warm (40 ± 2 °C) water. To evaluate the pork meat-induced facial expressions (neutral, happy, surprised, sad, scared, angry, contempt and disgusted), the recorded videos were analysed with the FaceReader software (Noldus Information Technology, Wageningen, the Netherlands). Only part of the video, when the panellists raised their hands, was used for the analysis of pork-meat-elicited emotions. The intensity of each emotion was expressed at a scale from 0 (no facial expression) to 1 (the highest intensity of facial expression). ‘Happy’ is considered as a positive emotion, whereas ‘sad’, ‘angry’, ‘scared’, ‘disgusted’ and ‘contempt’ are considered negative emotions. ‘Surprised’ can be either positive or negative. Valence indicated whether the person’s emotional status is positive or negative, and its score ranged from −1 to 1.

### 2.9. Statistical Analysis

The multivariate general linear model (GLM) was used for data analysis (SPSS for Windows, Ver.15.0, SPSS, Chicago, IL, USA). The *p*-values of factor interaction (experiment day × treatment type) were determined by tests of between-subjects effects in multivariate GLM. The mean values were compared using Duncan’s multiple range post hoc test with a significance level at *p* ≤ 0.05. Differences in bacterial genera between the groups were assessed using the Z-test calculator for two population proportions (Social Science Statistics, 2019). Statistical comparisons were considered significant when *p* ≤ 0.05.

## 3. Results and Discussion

### 3.1. Pigs’ Growth Performance

Data of the body weight (BW), average daily gain (ADG), feed intake (FI) and feed conversion ratio (FCR) of control (CG) and treated (TG-I) groups are presented in Table 2 The ADG of the 81–116 days of age period pigs was similar in both groups. However, during the experimental period (81–163 days of age), the ADG was lower in TG-I than that in CG. A similar trend was observed for BW, FI and FCR.

The feeds enriched with fibre ingredients have ambiguous effects on the growth performance of pigs, whereas high-fibre diets significantly reduce the ADG of weaned pigs [49]. The reason for the diminished growth performance of pigs can be related with the decrease in nutrient digestibility and energy deposition caused by dietary fibre [50]. Studies on ADG and FCR changes after SBP inclusion in pig diets have obtained inconsistent results. It was reported that the inclusion of SBP in pigs’ diet led to lower ADG [18,51]. However, Bruininx et al. [52] reported that including 10% of SBP into the diet increased the FCR during the growing period. Contrary, Millet et al. [53] found no significant differences in feed consumption, ADG or FCR when up to 20% SBP was introduced into the feed for pigs. It is likely that the different responses to diets containing various amounts of fibre are related to the age of the animals and the fibre amount and source [54]. In addition to SBP inclusion, other feed compounds and their interactions can be important factors influencing microbial profile, nutrients digestion, as well as BW, ADG and FCR results.

### 3.2. Microbial Profile of Pigs’ Faeces

**Figure 1:**
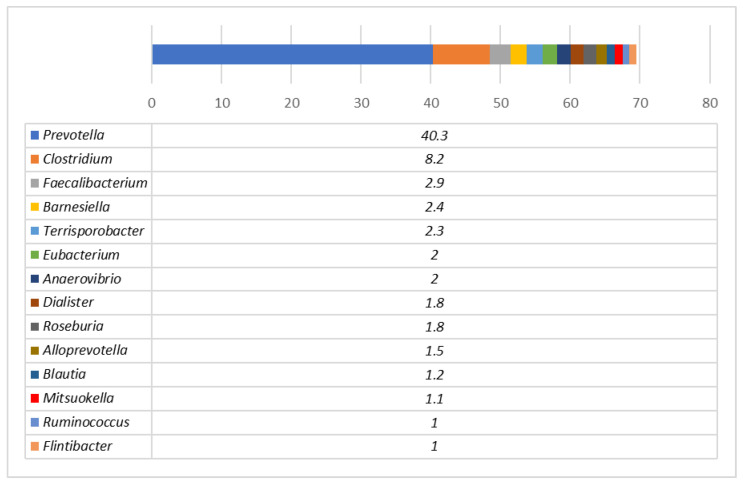
Microbial profiles of pig faeces before the experimental feeding (day 81). Genera with a prevalence of at least 1% from all bacterial reads are presented.

**Figure 2:**
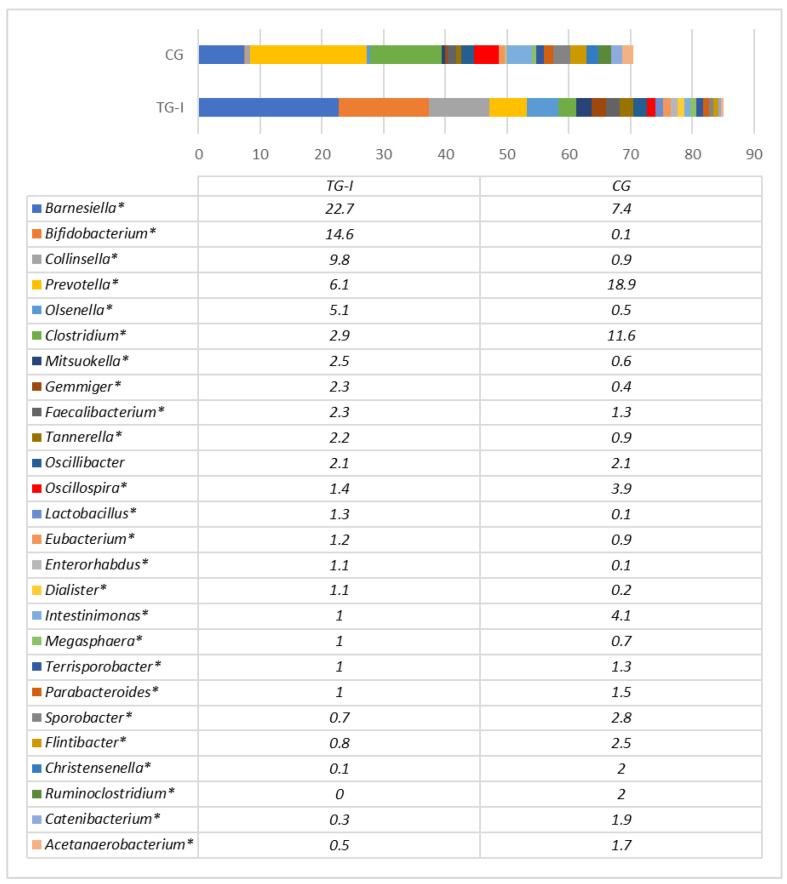
Microbial profiles of pigs’ faeces at genus level after experimental feeding (day 163). Bacterial genera with a prevalence of at least 1% from all bacterial reads in any of the tested groups are presented. * Statistically significant differences between groups.

Figure 1 shows the bacterial genera in the faeces of pigs before distribution into separate feeding groups. The most prevalent genera before the experimental feeding were *Prevotella* (40.3%) and *Clostridium* (8.2%). The remaining genera were *Faecalibacterium* (2.9%), *Barnesiella* (2.4%) and other bacterial genera that compose the core microbiota of pigs [55]. At the end of the experiment, the microbial profiles in pig faeces differed significantly (Figure 2). The most prevalent genera in the control group remained *Prevotella* (18.9%) and *Clostridium* (11.6%), followed by *Barnesiella* (7.4%), *Intestinimonas* (4.1%) and *Oscillospira* (3.9%), with a prevalence above 3% of the total bacteria. In the TG-I group, the most prevalent genera were *Barnesiella* (22.7%), *Bifidobacterium* (14.6%), *Collinsella* (9.8%), *Prevotella* (6.1%) and *Olsenella* (5.1%). The most obvious differences between the groups were the high (almost 150 times) increase in *Bifidobacterium* in the TG-I group and the less obvious decrease in *Clostridium* (4 times). *Bifidobacterium* contains various probiotic bacteria that produce lactic acid and can use a range of dietary carbohydrates that escape degradation in the upper parts of the intestine, many of which are plant-derived oligo- and polysaccharides [56]. It may be assumed that SBP had a strong influence on the proliferation of *Bifidobacterium* species that can use this carbohydrate-rich by-product. There is not much information about the significance of *Clostridium* for pigs, but it depends on the *Clostridium* species. Although some of the species can cause different diseases in pigs, a high prevalence of *Clostridium* was found in healthy pigs in this study; moreover, this genus is known as a constituent of normal microbiota of pigs and depends on pig age, breed and feed [57]. It is difficult to predict the importance of *Clostridium* and *Prevotella* decreases in the pig gut due to the significant increase in *Bifidobacterium*, and further studies are needed to evaluate the health status of pigs after such changes in microbial profiles.

Although the abundance of *Bifidobacterium* in pigs’ guts may depend on the feed [58], overall *Bifidobacterium*, contains species beneficial for pigs [59]. The other probiotic genera, *Lactobacillus*, was not highly abundant in both groups; the percental amount differed significantly between groups (*p* ≤ 0.05), with 13 times higher values (1.3% vs. 0.1%) in the TG-I group compared to the CG. The abundances of *Collinsella* and *Olsenella* were also higher in the TG-I group. *Collinsella* are short-chain FA producers feeding on animal- and plant-derived carbohydrates such as lactose, fructose, and starch, which are major components of the pigs’ diet [60,61]. The *Olsenella* is an effective carbohydrate fermenter and a producer of acetic and lactic acids; for these reasons, it is considered to be potentially beneficial [62]. The *Faecalibacterium* is known as probiotic bacterial genus [61]; it had a higher prevalence in the TG-I group, in comparison with control (2.3% vs. 1.3%; *p* ≤ 0.05). Finally, the faeces of pigs in the TG-I group contained significantly more bacteria that are considered as probiotic microbiota.

### 3.3. Blood Parameters

The blood parameters of pigs in CG and TG-I groups are given in Table 3. There was a significant effect of the SBP inclusion in pigs’ diet and animal age, as well as an interaction of these factors, on most blood parameters (*p* ≤ 0.05). Immunoglobulins IgM and IgG, ALB, and CREA concentrations were significantly lower (*p* ≤ 0.05) in the blood of 81- and 163-day-old pigs from TG-I group, in comparison with CG. The TP concentration was significantly (*p* ≤ 0.05) higher in the blood of 81-day-old pigs from TG-I but significantly lower in 163-day-old pigs in comparison with CG. The UREA concentration was significantly higher (*p* ≤ 0.05) in 163-day-old pigs from both groups compared to younger ones. The ALT showed significantly higher levels (*p* ≤ 0.05) in the blood of 81-day-old pigs from TG-I group, in comparison with CG. However, the ALT values of the blood from 163-day-old pigs from both groups were similar. Further, after 163 days, AST was significantly higher (*p* ≤ 0.05) in TG-I, in comparison with CG pigs’ blood. The values of AST and ALP were significantly reduced in older pigs, in comparison with younger ones, in both groups. The levels of ALP, CHOL, LDL, TGL, GLU, and T3 were significantly lower (*p* ≤ 0.05) in 163-day-old pigs from TG-I, in comparison with CG. The T4 concentration was significantly (*p* ≤ 0.05) higher in the blood of 163-day-old pigs of both groups, in comparison with younger ones. There were no significant differences between the groups in lgA, TSH, TBI, and HDL concentrations of 81- and 163-day-old pigs’ blood.

Blood biochemical parameters play a particularly important role in assessing the physiological and pathological conditions of animals [63]. Immunoglobulins (IgG, IgA, and IgM) are essential markers of the body’s humoral immunity and are present in the serum of all mammals [64]. Higher concentrations of IgG and IgM in serum are related with the increased antibody formation of B-lymphocytes [64]. Moreover, a higher concentration of IgM can be a sign of recent infections [65]. It was reported that SBP inclusion in weaning pigs’ diet is not significant factor on the levels of IgA and IgG in animals’ blood [5]. Perhaps greater concentrations of fibrous constituents are necessary to affect immunological outcomes [66]. Serum AST and ALT are indicators of liver function and health [66]. It was reported that an SBP diet was not significant on blood liver transaminases (ALT and AST), GLU and CREA of rabbits, and serum TP, TGL, and CHOL concentrations in rabbits blood were decreased [67]. However, studies about the SBP influence on pigs’ blood parameters are scarce. The lower concentration of TGL in TG-I pigs’ blood could be related to the higher levels of antioxidants in feed, reducing lipid peroxidation and potentially lowering TGL [68]. It can be concluded from the results that the inclusion of 3% of the SBP to the pigs’ diet is safe and does not impair animal metabolism, since all the blood parameters are in the normal range for pigs.

### 3.4. Carcass Parameters

Table 4 shows pig carcass grading and other carcass parameters of pigs from TG-I and CG, such as the muscularity, carcass weight, and weight average. Pigs fed with SBP had a significantly higher (*p* ≤ 0.05) percentage of carcasses in S and KN classes and a lower percentage (*p* ≤ 0.05) in E and U classes, compared to pigs in the CG. The SBP inclusion in pigs’ diet had no effect on the muscularity percentage in all classes of TG-I. The average carcass weight was significantly higher (*p* ≤ 0.05) in pigs of TG-I, compared to those of CG, except for class U. The diet of TG-I had no effect on the live weight average for class S, compared to the CG diet. However, the average live weight of E and KN classes was significantly higher (*p* ≤ 0.05) in TG-I, compared to CG. The carcass composition is influenced by the absorption, transformation, and deposition of energy. Dietary fibres reduce the energy intake and absorption in pigs, whereas long-term feeding with high fibre doses increases the intestinal weight [50]. However, the inconsistency of the effects of diets rich in dietary fibres on carcass properties can be explained by the breed, growth stage, and fibre level [50]. It was reported that pigs from SBP-fed gilts (*p* ≤ 0.05) had heavier carcass weights compared to control pigs (without SBP) [69]. Barakat et al. [65] revealed that the inclusion of 40% SBP with enzyme mixture in the rabbit diet significantly increased the final body weight and carcass %. However, Dunmire et al. [70] reported a decrease (*p* ≤ 0.10) in hot carcass weight and yield (*p* ≤ 0.05) in pigs fed SBP compared to those fed a control diet. Laitat et al. [71] reported a drop in carcass weight of 11% for pigs fed with 23% of SBP compared to a control group. Finally, the inclusion of 3% of the SBP to the pigs’ diet led to heavier carcass weights, and these findings can be related with the better digestibility of the feed nutrients, caused by the increased number of desirable bacteria in faeces microbial profile.

### 3.5. Meat Quality Parameters

#### 3.5.1. The Main Meat Quality Parameters

Table 5 shows the values of the main meat quality parameters of pigs fed with SBP (TG-I) and those without (CG). No significant differences in pH, dry matter, WHC, and ash content were found between TG-I and CG meat. The SBP incorporation into the pigs’ diet resulted in a higher DL and protein content but a lower CL and intramuscular fat content of the meat, compared to CG meat (*p* ≤ 0.05). Inclusion of SBP in pigs´ diet significantly affected values of lightness (L*), redness (a*), and yellowness (b*) coordinates of the tested meat. The meat of TG-I had a lower L* value and higher values of a* and b*, compared to the meat of CG.

The inclusion of fibre-rich ingredients in pigs’ diet can affect meat quality, although the observed findings are not consistent [25]. Studies on the influence of fibre-rich feed on meat quality are still scarce, and the mode of action is not clear. It was reported that the protein content of meat of the group fed with 75% SBP was significantly higher than that of the CG [72]. Additionally, the SBP diet decreased the CL (*p* ≤ 0.01) compared to the meat in the CG [73]. Joven et al. [74] and Li et al. [75] revealed that the inclusion of fibre-rich ingredients in the diet decreased backfat thickness in gilts and finishing pigs. However, it was reported that 50% of SBP inclusion significantly increased the fat content in meat [72,76]. Moreover, Qin et al. [61] found that the moisture of lamb meat was not affected by SBP supplementation. The values of the colour coordinates observed during this study were similar to those reported by other authors [72,77,78]. The colour of pork meat is determined by the distribution and amounts of deoxymyoglobin, oxymyoglobin, and metmyoglobin, as well as the structure and physical state of muscle proteins and the proportion of intramuscular fat [79]. The decrease in a* value could be related to an increase in metmyoglobin, whereas the b* value increases with the increase in oxymyoglobin [80]. Thus, an increase in the amount of intramuscular fat in meat can lead to a decrease in the concentrations of myoglobin and sarcoplasmic proteins, leading to changes in meat colour [81]. The inclusion of certain nutrients (vitamins and minerals) into the diet or diets low in digestible carbohydrates affect pork colour, whereas most studies found no effect of those diets on meat colour [82]. Finally, the inclusion of 3% of the SBP to the pigs’ diet resulted in a higher DL and protein content, lower L* value and higher values of a* and b* coordinates of the meat. 

#### 3.5.2. Biogenic Amine Contents and Fatty Acid Profiles of Pork Meat

The BA contents in the meat of pigs from CG and TG-I groups are given in Table 6. No BAs, except spermine, were found in all tested samples from both groups, indicating that SBP inclusion in the diet had no significant impact on the spermine content in both groups.

Endogenic BAs are excreted by different tissues in humans or animals and transferred locally or via the blood system, whereas exogenic BAs are formed by the action of decarboxylase-positive microorganisms on free amino acids [83]. Usually, the BA level is considered an index for food product stability and quality because high contents of these compounds negatively affect human health [84]. The presence of spermine in the tested meat of both groups is related with the higher amount of this BA in fresh meat (20–60 mg/kg) [85]. Similar tendencies have been observed in other studies [86,87]. Finally, the inclusion of 3% of the SBP to the pigs’ diet was not significant on BA formation in meat. 

#### 3.5.3. The Fatty Acid Profile of Pork Meat

The FA profile of pork meat from CG and TG-I groups is shown in Table 7. Saturated FAs (SFAs) were the most abundant (42–43%) in both groups due to the high levels of C-16:0 (23–24%) and C-18:0 (15–16%). The proportion of monounsaturated FAs (MUFA) was 36–42%; the most important FA were C-18:1 (30–38%). Polyunsaturated FA (PUFA) accounted for 14–22%, including mainly C-18:2 (11–18%). Significant differences in the amounts of certain FA, SFA, MUFA, and PUFA among the tested groups were found (*p* ≤ 0.05). The amounts of C14:0, C16:1, C17:0, C17:1, C18:2, C18:3 α, C20:0, C20:1, C20:2, C20:3, and C20:4 FA were significantly higher, namely by 37.4, 20%, 2-fold, 1.9-fold, 55.7, 18.4, 28.4, 25.7, 43.8, 57.1%, and 2.1-fold, respectively, in pork meat from the TG-I group, compared to the CG group (*p* ≤ 0.05). Significantly lower amounts of total SFAs and MUFA were determined in the meat of the TG-I group, whereas in this group, the total PUFA amount was 51.3% higher compared to CG meat. 

Tissue FA biosynthesis and FA composition obtained from the diet influence the FA profile of pork meat [88]. Pigs are monogastrics, and the microbiota of their digestive tract does not metabolise FAs prior absorption [89]. Opposite to SFA and MUFA, linoleic and α-linolenic FA cannot be synthesized in the body of pigs, and their levels in pork meat are related to the percentages of them found in the diet [78]. In the present study, the amounts of these FAs were higher in meat from pigs fed with SBP. It was reported that higher amounts of linoleic (C18:2) and myristic acid (C14:0) and a lower content of oleic acid (C18:1) are in the abdominal fat of geese fed a pressed SBP-silage-supplemented diet [90].

#### 3.5.4. Volatile Compound Profile of Pork Meat

The VCs of pork meat from CG and TG-I groups are given in Table 8. The main VCs in pork meat were hexanal, followed by nonanal and 2-methyl-3-octanone. The meat of pigs fed with SBP-supplemented diet (TG-I) had significantly higher (*p* ≤ 0.05) contents of 2-methyl-3-octanone, 2-pentyl-furan, and (E,E)-2,4-nonadienal compared to the meat of CG. In the meat of the CG, the contents of nonanal, benzothiazole, tridecane, tridecanal, and 2,4-bis(1,1-dimethylethyl)phenol were significantly higher (*p* ≤ 0.05) compared to meat of the TG-I. Moreover, heptanal, (E)-2-heptenal, benzyl alcohol, oct-3-en-2-one, cyclooctyl alcohol, nonan-3-one, and dec-(4Z)-enal were not found in the meat of CG, whereas these VCs occurred in the meat of TG-I. The concentrations of other identified VCs were similar for both groups.

Although raw meat has a weak odour, the flavour is formed due to many factors, including the degradation of proteins to amino acids, the transformation of glycogen to glucose, and the autoxidation of the lipid fraction [91]. Animal feed composition, especially the FA profile, is relevant for the formation of VCs such as ketones, acids, aldehydes and alcohols [92]. Aldehydes, such as hexanal, heptanal, nonanal, and decanal, may occur due to lipid oxidation in pork samples [93]. Hexanal and nonanal, with fresh, fatty, grassy and fruity notes, were the main compounds that created the overall aroma in pork meat of the CG group [94]. The higher contents of these compounds, together with 2-methyl-3-octanone, a methyl ketone, contributed to the aroma of meat of the TG-I group. Methyl ketones, with fatty notes, are formed due to secondary lipid oxidation [95]. It was reported that lipid oxidation causes alcohol production, whereas lipid autoxidation and microbiological metabolism promote the formation of ketones [96]. Unsaturated aldehydes, such as (E,E)-2,4-nonadienal, are degradation products of linoleate and linolenate hydroperoxides [93].

#### 3.5.5. Pork Meat Sensory Properties, Overall Acceptability, and Emotions Induced in the Judges

The sensory properties of pork meat from CG and TG-I groups are given in Table 9. The scores for colour, fattiness, residual, and overall taste intensiveness, as well as overall acceptability, were significantly higher (*p* ≤ 0.05) for the TG-I group meat. The softness, juiciness and odour (overall and extraneous) were similar for both meat groups. The emotional responses elicited by the tested meat are given in Table 10. The facial expression ‘neutral’ was predominant in both meat groups, and its intensity was the highest, compared to other facial expressions. The intensities of the facial expressions ‘happy’, ‘surprised’, ‘scared’, ‘disgusted’, and ‘contempt’ were significantly higher (*p* ≤ 0.05) for meat of the TG-I group. For the remaining emotions (neutral, sad, and angry), the values of intensiveness were similar for both groups. 

Many intrinsic factors both pre- and post-slaughtering have influences on the sensory rating of meat [97]. For the most part, the liking variability of meat could be explained by colour and flavour properties [97]. This was also observed in the present study, because colour and taste were rated higher in most liked samples. The higher rating of flavour intensiveness of TG-I meat might be related to the higher production of VC from oxidised FA [78]. Similar to our results, other researchers have reported that sensory properties (flavour, odour, texture) and the overall acceptability of lamb, beef, and pork meat were influenced by the animal diet [98,99,100]. Moreover, Diaz et al. [78] found that the odour, flavour, texture, and overall score of dry-cured sausage from the meat from pigs fed dried chestnuts and an SBP-supplemented diet were significantly higher rated, than those of the CG.

The sensory properties of food could elicit different emotional reactions for consumers [101]. Nowadays, the evaluation of emotional responses plays an essential part in the field of sensory science because it reflects the stronger relationship between consumers and food products, compared to hedonic reactions [97]. Kostyra et al. [101] used FaceReader software to measure the facial expressions of participates provoked by the consumption of smoked hams. The ham samples elicited various emotional reactions, of which neutral and negative emotions were predominant. Finally, despite the fact that the intensities of the facial expressions (in addition to ‘happy’) ‘surprised’, ‘scared’, ‘disgusted’, and ‘contempt’ were significantly higher for meat of the TG-I group, the valence values of both groups were similar, and higher overall acceptability was shown by TG-I meat samples. 

## 4. Conclusions

The 3% of SBP inclusion to the pig diet is safe (all the blood parameters were in the normal range); however, it reduced the BW and ADG of animals but improved carcass quality. Desirable changes in the TG-I group may be related to the higher levels of beneficial bacteria in their faecal profile. The tested diet was not significant on BA formation in pork, and the main VC in meat were hexanal, followed by nonanal and 2-methyl-3-octanone. The SBP inclusion to pigs’ diet reduces the total SFAs and MUFAs content, as well as increases the meat drip loss, protein content, and redness. The valence values of both groups were similar; however, higher overall acceptability was shown by TG-I meat samples. Finally, it can be stated that 3% of SBP inclusion to the diet of pigs could be beneficial for the improvement of pigs’ gut health and pork quality. However, further studies are needed to indicate which compounds of the SBP dietary fiber are responsible for these desirable changes.

## Figures and Tables

**Table 1:** Diet composition of control (CG) and treated (TG-I) groups.

Ingredients (%)	CG	TG-I	CG	TG-I
	First Fattening Period (81–116 Days)		Second Fattening Period (116–163 Days)	
Barley	69.90	66.90	63.00	60.00
Barley malt sprouts	-	-	6.50	6.50
Grain mix (barley-buckwheat 60:40)	-	-	6.79	6.79
Oats	3.00	3.00	5.00	5.00
Rape meal	-	-	6.00	6.00
Wheat bran	5.00	5.00	6.00	6.00
Hulled soybean meal GMO	7.20	7.20	2.87	2.87
Dried sugar beet pulp	-	3.00	-	3.00
Molasses	0.57	0.57	-	-
Rapeseed oil unrefined	1.91	1.91	-	-
Peas	3.00	3.00	-	-
Rape seed expeller	5.00	5.00	-	-
NaCl	0.35	0.35	0.35	0.35
Calcium carbonate	1.38	1.38	1.18	1.18
Monocalcium phosphate	0.39	0.39	0.32	0.32
DL-Methionine	0.19	0.19	0.04	0.04
Biolys	0.92	0.92	0.65	0.65
Adici FP liquid	0.15	0.15	0.25	0.25
Choline chloride 75% liquid	0.04	0.04	0.02	0.02
Vitamins and trace elements (premix)	1.00 ^ii^	1.00 ^ii^	1.00 ^iii^	1.00 ^iii^
*Nutritional value*				
ME swine (MJ/kg)	12.83	12.83	11.97	11.97
Crude protein (%)	17.00	17.00	15.00	15.00
Crude fat (%)	5.01	5.01	2.93	2.93
Crude fibre (%)	4.85	4.85	5.79	5.79
Lysine (%)	1.13	1.13	0.89	0.89
Methionine (%)	0.41	0.41	0.26	0.26
Threonine (%)	0.73	0.73	0.58	0.58
Tryptophan (%)	0.20	0.20	0.17	0.17
Methionine + Cystine (%)	0.68	0.68	0.54	0.54
Ca (%)	0.70	0.70	0.67	0.67
Total P (%)	0.51	0.51	0.53	0.53
Available P (%)	0.25	0.25	0.25	0.25
Na (%)	0.17	0.17	0.18	0.18
NDF (%)	14.82	14.95	15.75	15.95
ADF (%)	4.88	4.98	5.02	5.12

(^ii^) basal diet with dried sugar beet pulp (TG-I); ME—metabolisable energy; (^iii^) Composition of premix per 1 kg of feed: Vitamin A—8000 IU; vitamin D3—1500 IU; vitamin E—100 mg/kg; vitamin K3—3.25 mg; thiamine—2.06 mg; riboflavin—4.00 mg; choline chloride—300 mg; pyridoxine—3.07 mg; vitamin B12—0.03 mg; niacin—20.0 mg; pantothenic acid—25.00 mg; folic acid—0.95 mg; biotin—0.11 mg; Fe—120 mg; Cu—20 mg; Zn—102 mg; Mn—40 mg; I—1.46 mg; Co—0.52 mg; Se—0.40 mg. NSP Enzyme, Rovabio Excel AP, 50 g/t; endo-1,4-β-xylanase 1100 VU /kg of feed; endo-1,3 (4)-glucanase, 1500 VU/kg of feed and Phytase Axtra PHY 10,000 TPT 2, 130 g/t, 1300 FTU/kg feed; (iv) Composition of premix per 1 kg of feed: Vitamin A—7000 IU; vitamin D3—2000 IU; vitamin E—70 mg/kg; vitamin K3—1.19 mg; thiamine—1.18 mg; riboflavin—2.32 mg; choline chloride—150 mg; pyridoxine—2.28 mg; vitamin B12—0.03 mg; niacin—14.03 mg; pantothenic acid—11.50 mg; folic acid—0.57 mg; biotin—0.13 mg; Fe—100 mg; Cu—15 mg; Zn—101 mg; Mn—46 mg; I—0.80 mg; Co—0.57 mg; Se—0.40 mg. NSP Enzyme, Rovabio Excel AP, 50 g/t; endo-1,4-β-xylanase 1 100 VU /kg of feed; endo-1,3 (4)-glucanase, 1500 VU/kg of feed and Phytase Axtra PHY 10,000 TPT 2, 130 g/t, 1300 FTU/kg feed. Biolys—L-Lysin sulphate, L-Lysine 55.6%.

**Table 2:** Pigs’ growth performance in control (CG) and treated (TG-I) groups.

Growth Parameters	Period	CG	TG-I
Body weight (kg)	81	24.85 ± 0.15 ^a^	25.20 ± 0.40 ^a^
	116	43.24 ± 0.52 ^a^	43.70 ± 0.34 ^a^
	163	95.94 ± 0.85 ^a^	94.30 ± 0.93 ^a^
ADG (kg)	81–116	0.52 ^a^	0.53 ^a^
	117–163	1.14 ^a^	1.10 ^a^
	81–163	0.88 ^a^	0.85 ^a^
FI (kg)	81–116	1.28 ^a^	1.33 ^a^
	117–163	3.58 ^a^	3.05 ^a^
	81–163	2.46 ^a^	2.24 ^a^
Feed Conversion Ratio (FCR, 1 kg of feed per 1 kg of body weight)	81–116	2.45 ^a^	2.50 ^a^
	117–163	3.14 ^b^	2.77 ^a^
	81–163	2.80 ^b^	2.64 ^a^

Basal diet (CG) and basal diet with dried sugar beet pulp (TG-I). Data are presented as mean ± standard error (*n* = 150/group). ^a,b^ Different letters indicate significant differences between treatments (*p* ≤ 0.05).

**Table 3:** Blood parameters of pigs in control (CG) and treated (TG-I) groups.

Blood Parameters	Day	CG	TG-I	*p* Day × Treatment Interaction
IgA	81	0.330 ± 0.011 ^Aa^	0.330 ± 0.014 ^Aa^	1
	163	0.330 ± 0.013 ^Aa^	0.330 ± 0.012 ^Aa^	
IgM	81	0.816 ± 0.027 ^Ab^	0.544 ± 0.017 ^Aa^	<0.001
	163	1.066 ± 0.017 ^Bb^	0.928 ± 0.023 ^Ba^	
IgG	81	3.492 ± 0.054 ^Ab^	3.310 ± 0.039 ^Aa^	<0.001
	163	4.134 ± 0.067 ^Bb^	3.362 ± 0.041 ^Aa^	
TTH	81	0.014 ± 0.003 ^Aa^	0.018 ± 0.004 ^Aa^	<0.001
	163	0.014 ± 0.002 ^Aa^	0.012 ± 0.001 ^Aa^	
ALB	81	36.60 ± 0.57 ^Ab^	34.16 ± 0.23 ^Aa^	0.412
	163	42.00 ± 0.36 ^Bb^	38.60 ± 0.32 ^Ba^	
TP	81	48.00 ± 0.41 ^Aa^	52.40 ± 0.48 ^Ab^	<0.001
	163	62.00 ± 0.56 ^Bb^	57.60 ± 0.51 ^Ba^	
UREA	81	2.50 ± 0.21 ^Aa^	2.26 ± 0.18 ^Aa^	0.031
	163	3.58 ± 0.37 ^Ba^	3.62± 0.28 ^Ba^	
CREA	81	78.10 ± 1.45 ^Ab^	64.38 ± 2.14 ^Aa^	<0.001
	163	121.60 ± 4.27 ^Bb^	90.50 ± 3.42 ^Ba^	
ALT	81	56.80 ± 2.36 ^Aa^	67.00 ± 3.51 ^Ab^	0.002
	163	67.60 ± 1.62 ^Ba^	68.60 ± 2.43 ^Aa^	
AST	81	30.60 ± 2.39 ^Ba^	30.40 ± 2.07 ^Ba^	0.009
	163	24.20 ± 1.14 ^Aa^	26.80 ± 1.25 ^Ab^	
ALP	81	216.80 ± 1.40 ^Bb^	213.80 ± 1.09 ^Ba^	0.025
	163	178.40 ± 5.62 ^Ab^	158.80 ± 3.72 ^Aa^	
TBI	81	3.00 ± 0.04 ^Aa^	3.00 ± 0.02 ^Aa^	1
	163	3.00 ± 0.03 ^Aa^	3.00 ± 0.01 ^Aa^	
CHOL	81	2.60 ± 0.07 ^Aa^	2.61 ± 0.09 ^Ba^	0.002
	163	2.67 ± 0.11 ^Ab^	2.37 ± 0.08 ^Aa^	
DTL	81	1.06 ± 0.12 ^Aa^	1.15 ± 0.09 ^Aa^	0.002
	163	1.07 ± 0.14 ^Aa^	1.03 ± 0.11 ^Aa^	
MTL	81	1.30 ± 0.18 ^Aa^	1.18 ± 0.09 ^Aa^	0.001
	163	1.76 ± 0.12 ^Bb^	1.42 ± 0.11 ^Ba^	
TGL	81	0.520 ± 0.050 ^Aa^	0.620 ± 0.059 ^Ba^	<0.001
	163	0.720 ± 0.032 ^Bb^	0.380 ± 0.029 ^Aa^	
GLU	81	5.36 ± 0.26 ^Aa^	5.78 ± 0.31 ^Ba^	<0.001
	163	5.42 ± 0.42 ^Ab^	4.28 ± 0.23 ^Aa^	
T3	81	1.43 ± 0.11 ^Aa^	2.02 ± 0.18 ^Bb^	<0.001
	163	1.78 ± 0.09 ^Bb^	1.10 ± 0.08 ^Aa^	
T4	81	3.00 ± 0.23 ^Aa^	2.97 ± 0.09 ^Aa^	0.913
	163	3.54 ± 0.15 ^Ba^	3.52 ± 0.21 ^Ba^	

Basal diet (CG), basal diet with dried sugar beet pulp (TG-I); IgA, IgM, IgG- immunoglobulin, g/L; TTH—thyroid-stimulating hormone; ALB—albumin, g/L; TP—total protein, g/L); UREA—urea, mmol/L; CREA—creatinine, µmol/L; ALT—alanine aminotransferase, U/L; AST—aspartate aminotransferase, U/L; ALP—alkaline phosphatase, U/L; TBI—total bilirubin, pmol/L; CHOL—cholesterol, mmol/L; DTL—high-density lipoprotein cholesterol, mmol/L; MTL—low-density lipoprotein cholesterol, mmol/L; TGL—triglycerides, mmol/L; GLU—glucose, nmol/L; T3—triiodothyronine, nmol/L; T4—thyroxine, µd/L. Data are presented as mean ± standard error (*n* = 15/group). ^A,B^ Different capitals indicate significant time-related differences (*p* ≤ 0.05); ^a,b^ different letters indicate significant differences between treatments (*p* ≤ 0.05).

**Table 4:** Carcass parameters of piglets in control (CG) and treated (TG-I) groups.

Carcass Parameters	Classes	CG	TG-I
Classes, %	S	59.40 ± 1.26 ^a^	84.00 ± 1.31 ^b^
	E	37.20 ± 2.26 ^b^	12.80 ± 0.24 ^a^
	U	2.80 ± 0.21 ^b^	1.10 ± 0.08 ^a^
	KN	0.60 ± 0.05 ^a^	1.70 ± 0.14 ^b^
Muscularity, average, %	S	62.03 ± 1.38 ^a^	63.24 ± 2.07 ^a^
	E	58.45 ± 1.14 ^a^	58.32 ± 1.95 ^a^
	U	53.82 ± 1.96 ^a^	53.60 ± 1.21 ^a^
	KN	77.10 ± 1.72 ^a^	77.03 ± 1.58 ^a^
Carcass weight average, kg	S	84.07 ± 0.65 ^a^	85.62 ± 0.72 ^b^
	E	86.97 ± 1.26 ^a^	90.93 ± 0.89 ^b^
	U	91.06 ± 0.63 ^b^	90.01 ± 0.57 ^a^
	KN	104.00 ± 2.26 ^a^	109.17 ± 1.34 ^b^
Live weight average, kg	S	113.45 ± 2.26 ^a^	113.53 ± 1.84 ^a^
	E	117.36 ± 1.03 ^a^	120.00 ± 1.12 ^b^
	U	122.80 ± 1.14 ^b^	119.00 ± 1.05 ^a^
	KN	107.12 ± 2.51 ^a^	144.47 ± 1.38 ^b^

Basal diet (CG), basal diet with dried sugar beet pulp (TG-I); KN—non-evaluated carcass. Data are presented as mean ± standard error (*n* = 150/group). ^a,b^ Different letters indicate significant differences between treatments (*p* ≤ 0.05).

**Table 5:** Main meat quality parameters of piglets in control (CG) and treated (TG-I) groups.

Quality Parameters		CG	TG-I
Dry matter, %		28.05 ± 0.21 ^a^	28.28 ± 0.23 ^a^
pH		5.59 ± 0.03 ^a^	5.54 ± 0.02 ^a^
Colour coordinates	L*	62.92 ± 0.31 ^b^	60.28 ± 0.27 ^a^
	a*	14.91 ± 0.11 ^a^	17.58 ± 0.10 ^b^
	b*	10.10 ± 0.09 ^a^	12.65 ± 0.08 ^b^
Drip loss, %		3.45 ± 0.12 ^a^	3.77 ± 0.17 ^b^
Water holding capacity, %		57.67 ± 0.91 ^a^	58.19 ± 0.56 ^a^
Cooking loss, %		26.33 ± 0.25 ^b^	25.09 ± 0.23 ^a^
Intramuscular fat, %		5.56 ± 0.04 ^b^	4.66 ± 0.03 ^a^
Ash, %		1.19 ± 0.02 ^a^	1.21 ± 0.01 ^a^
Protein, %		21.33 ± 0.18 ^a^	22.41 ± 0.21 ^b^

Basal diet (CG), basal diet with dried sugar beet pulp (TG-I). L*, lightness; a*, redness or -a*, greenness; b*, yellowness or -b*, blueness. Data are presented as mean ± standard error (*n* = 10/group). ^a,b^ Different letters indicate significant differences between treatments (*p* ≤ 0.05).

**Table 6:** Biogenic amine contents of pork meat.

Biogenic Amines, mg/kg	Control Group (CG)	TG-I
Tryptamine	nd	nd
Phenylethylamine	nd	nd
Putrescine	nd	nd
Cadaverine	nd	nd
Histamine	nd	nd
Tyramine	nd	nd
Spermidine	nd	nd
Spermine	64.98 ± 3.21	64.83 ± 2.79

Basal diet (CG), basal diet with dried sugar beet pulp (TG-I); nd—not detected. Data are presented as mean ± standard error (*n* = 10/group).

**Table 7:** Fatty acid profile (percentage of the total fatty acid methyl esters concentration in pork meat).

Fatty Acids		CG	TG-I
C4:0	Butyric acid	nd	nd
C6:0	Hexanoic acid	nd	nd
C8:0	Octanoic acid	nd	nd
C10:0	Decanoic acid	nd	0.025 ± 0.01
C11:0	Undecanoic acid	nd	nd
C12:0	Lauric acid	nd	0.011 ± 0.01
C13:0	Tridecanoic acid	nd	nd
C14:0	Tetradecanoic acid	1.74 ± 0.02 ^a^	2.39 ± 0.01 ^b^
C14:1	Myristoleic acid	nd	nd
C15:0	Pentadecanoic acid	nd	0.071 ± 0.01
C15:1	*cis*-10-pentadecenoic acid	nd	nd
C16:0	Palmitic acid	24.8 ± 0.1 ^b^	23.2 ± 0.1 ^a^
C16:1	Palmitoleic acid	3.35 ± 0.02 ^a^	4.02 ± 0.03 ^b^
C17:0	Heptadecanoic acid	0.346 ± 0.002 ^a^	0.722 ± 0.003 ^b^
C17:1	*cis*-10-heptadecanoic acid	0.289 ± 0.002 ^a^	0.544 ± 0.003 ^b^
C18:0	Stearic acid	15.9 ± 0.1 ^a^	15.7 ± 0.2 ^a^
C18:1 *cis,trans*	*all cis,trans*-9-octadecenoic acid	38.0 ± 0.2 ^b^	30.03 ± 0.1 ^a^
C18:2	Linoleic acid	11.5 ± 0.1 ^a^	17.9 ± 0.2 ^b^
C18:2 *trans*	Linolelaidic acid	nd	nd
C18:3 γ	γ- linolenic acid	nd	0.020 ± 0.01
C18:3 α	α—linolenic acid	1.47 ± 0.02 ^a^	1.74 ± 0.03 ^b^
C20:0	eicosanoic acid	0.225 ± 0.003 ^a^	0.289 ± 0.004 ^b^
C20:1	*cis*-11-eicosenoic acid	1.13 ± 0.02 ^a^	1.42 ± 0.01 ^b^
C20:2	*cis*-11,14-eicosadienoic acid	0.543 ± 0.003 ^a^	0.781 ± 0.004 ^b^
C20:3	*cis*-8,11,14-eicosatrienoic acid	0.149 ± 0.001 ^a^	0.234 ± 0.002 ^b^
C21:0	Heinecosonoic acid	nd	nd
C20:4	*cis*-5,8,11,14-eicosatetraenoic acid	0.283 ± 0.002 ^a^	0.604 ± 0.003 ^b^
C20:3	*cis*-11,14,17-eicosatrienoic acid	0.263 ± 0.004 ^b^	0.252 ± 0.002 ^a^
C20:5	*cis*-5,8,11,14,17-eicosapentanoic acid	nd	nd
C22:0	Docosanoic acid	nd	nd
C22:1	*cis*-13-docosenoic acid	nd	nd
C22:2	*cis*-13,16-docosadienoic acid	nd	nd
C23:0	Tricosanoic acid	nd	nd
C24:0	Tetracosanoic acid	nd	nd
C22:6	*all cis*-4,7,10,13,16,19-docosahexanoic acid	nd	nd
C24:1	*cis*-15-tetracosenoic acid	nd	nd
Saturated fatty acids		43.04 ^b^	42.44 ^a^
Monounsaturated fatty acids		42.72 ^b^	36.01 ^a^
Polyunsaturated fatty acids		14.24 ^a^	21.55 ^b^

Basal diet (CG), basal diet with dried sugar beet pulp (TG-I); nd—not detected. Data are presented as mean ± standard error (*n* = 10/group). ^a,b^ Different letters indicate significant differences between treatments (*p* ≤ 0.05).

**Table 8:** Volatile compound (percentage of the total volatile compounds content) profiles of pork meat from control and treated groups.

Retention Time, min	Volatile Compound	CG	TG-I
5.722	Hexanal	37.90 ± 6.28 ^a^	38.7 ± 5.08 ^a^
8.384	Heptanal	nd	0.34 ± 0.12
9.840	(E)-2-Heptenal	nd	0.68 ± 0.60
9.926	Benzaldehyde	1.42 ± 1.28 ^a^	2.59 ± 0.52 ^a^
10.195	1-Heptanol	0.48 ± 0.46 ^a^	1.21 ± 1.05 ^a^
10.448	1-Octen-3-ol	5.73 ± 0.70 ^a^	6.89 ± 1.08 ^a^
10.560	2-methyl-3-Octanone	9.74 ± 0.56 ^a^	19.10 ± 4.44 ^b^
10.783	2-pentyl-furan	1.27 ± 1.11 ^a^	3.42 ± 0.33 ^b^
11.730	2-ethyl-1-hexanol	3.28 ± 1.75 ^a^	1.23 ± 0.91 ^a^
11.865	Benzyl alcohol	nd	0.31 ± 0.15
11.961	Oct-3-en-2-one	nd	0.14 ± 0.05
12.440	(E)-2-Octenal	2.47 ± 0.24 ^a^	2.28 ± 0.53 ^a^
12.688	Cyclooctyl alcohol	nd	0.80 ± 0.16
12.745	1-Octanol	2.95 ± 0.98 ^a^	2.02 ± 0.17 ^a^
13.150	Nonan-3-one	nd	0.39 ± 0.04
13.576	Nonanal	20.2 ± 3.52 ^b^	11.1 ± 0.12 ^a^
14.888	(E)-2-Nonenal	0.94 ± 0.21 ^a^	0.65 ± 0.18 ^a^
15.068	Octanoic acid	0.74 ± 0.33 ^a^	0.60 ± 0.11 ^a^
15.684	Dec-(4 Z)-enal	nd	0.05 ± 0.09
15.805	Dodecane	0.10 ± 0.07	nd
15.936	Decanal	0.53 ± 0.16 ^a^	0.34 ± 0.11 ^a^
16.135	(E,E)-2,4-nonadienal	0.20 ± 0.04 ^a^	0.41 ± 0.03 ^b^
16.494	Benzothiazole	1.09 ± 0.29 ^b^	0.17 ± 0.30 ^a^
17.017	Geraniol	0.35 ± 0.12 ^a^	0.19 ± 0.16 ^a^
17.177	Dec-(2E)-enal	0.95 ± 0.83 ^a^	0.59 ± 0.52 ^a^
17.215	Nonanoic acid	1.60 ± 1.54 ^a^	0.65 ± 0.49 ^a^
17.576	Tridecane	1.47 ± 0.25 ^b^	0.78 ± 0.13 ^a^
17.877	(E,E)-2,4-dodecadienal	0.58 ± 0.06 ^a^	0.48 ± 0.07 ^a^
17.961	Indole	0.42 ± 0.38 ^a^	0.33 ± 0.08 ^a^
18.366	Deca-(2E,4E)-dienal	1.21 ± 0.12 ^a^	1.03 ± 0.08 ^a^
19.181	Propanoic acid, 2-methyl-, 2,2-dimethyl-1-(2-hydroxy-1-methylethyl)propyl ester	0.24 ± 0.23 ^a^	0.23 ± 0.09 ^a^
19.245	n-Decanoic acid	0.11 ± 0.19 ^a^	nd
19.317	2-Undecenal	1.44 ± 0.62 ^a^	0.96 ± 0.12 ^a^
20.021	Tetradecane	0.40 ± 0.35 ^a^	0.28 ± 0.10 ^a^
21.484	2,6-bis(1,1-dimethylethyl)-2,5-cyclohexadiene-1,4-dione	0.19 ± 0.12 ^a^	0.28 ± 0.18 ^a^
22.159	Tridecanal	0.51 ± 0.07 ^b^	0.27 ± 0.13 ^a^
22.216	2,4-bis(1,1-dimethylethyl)phenol	0.64 ± 0.28 ^b^	0.08 ± 0.05 ^a^
24.000	Tetradecanal	0.87 ± 0.26 ^a^	0.54 ± 0.12 ^a^

Basal diet (CG), basal diet with dried sugar beet pulp (TG-I); nd—not detected. Data are presented as mean ± standard error (*n* = 10/group). ^a,b^ Different letters indicate significant differences between treatments (*p* < 0.05).

**Table 9:** Sensory properties and overall acceptability of pork meat from control and treated groups.

Sensory Properties	CG	TG-I
Overall odour	2.55 ± 0.27 ^a^	2.42 ± 0.30 ^a^
Extraneous odour	1.05 ± 0.11 ^a^	1.08 ± 0.13 ^a^
Colour	2.50 ± 0.25 ^a^	3.08 ± 0.13 ^b^
Juiciness	2.10 ± 0.42 ^a^	2.00 ± 0.45 ^a^
Fattiness	1.75 ± 0.35 ^a^	2.58 ± 0.79 ^b^
Softness	2.25 ± 0.35 ^a^	2.38 ± 0.59 ^a^
Residual taste	1.00 ± 0.10 ^a^	2.75 ± 0.42 ^b^
Overall taste	2.70 ± 0.48 ^a^	3.13 ± 0.26 ^b^
Overall acceptability	2.65 ± 0.29 ^a^	3.08 ± 0.34 ^b^

Basal diet (CG), basal diet with dried sugar beet pulp (TG-I). Data are presented as mean ± standard error (*n* = 10/group). ^a,b^ Different letters indicate significant differences between treatments (*p* ≤ 0.05).

**Table 10:** Emotional responses elicited by pork meat consumption.

Facial Expression	CG	TG-I
Neutral	0.712 ± 0.132 ^a^	0.814 ± 0.158 ^a^
Happy	0.028 ± 0.03 ^a^	0.041 ± 0.06 ^b^
Sad	0.044 ± 0.06 ^a^	0.039 ± 0.05 ^a^
Angry	0.028 ± 0.04 ^a^	0.030 ± 0.03 ^a^
Surprised	0.017 ± 0.002 ^a^	0.047 ± 0.005 ^b^
Scared	0.004 ± 0.002 ^a^	0.032 ± 0.004 ^b^
Disgusted	0.014 ± 0.003 ^a^	0.041 ± 0.005 ^b^
Contempt	0.004 ± 0.002 ^a^	0.033 ± 0.004 ^b^
Valence	0.060 ± 0.009 ^a^	0.057 ± 0.011 ^a^

Basal diet (CG), basal diet with dried sugar beet pulp (TG-I). Data are presented as mean ± standard error (*n* = 10/group). ^a,b^ Different letters indicate significant differences between treatments (*p* ≤ 0.05).

## Data Availability

Not applicable.
